# NeuroD2 regulates the development of hippocampal mossy fiber synapses

**DOI:** 10.1186/1749-8104-7-9

**Published:** 2012-02-27

**Authors:** Scott A Wilke, Benjamin J Hall, Joseph K Antonios, Laura A DeNardo, Stefanie Otto, Bo Yuan, Fading Chen, Elissa M Robbins, Katie Tiglio, Megan E Williams, Zilong Qiu, Thomas Biederer, Anirvan Ghosh

**Affiliations:** 1 Neurobiology Section, Division of Biological Sciences, University of California, San Diego, CA 92093-0366, USA; 2 Department of Cell and Molecular Biology, Tulane University, New Orleans, LA 70118, USA; 3 Institute of Neuroscience, Shanghai Institute of Biological Sciences, Chinese Academy of Sciences, Shanghai, PR China; 4 Department of Molecular Biophysics and Biochemistry, Yale University, New Haven, CT 06520, USA; 5 Department of Neurobiology and Anatomy, University of Utah, Salt Lake City, UT 84132, USA

## Abstract

**Background:**

The assembly of neural circuits requires the concerted action of both genetically determined and activity-dependent mechanisms. Calcium-regulated transcription may link these processes, but the influence of specific transcription factors on the differentiation of synapse-specific properties is poorly understood. Here we characterize the influence of NeuroD2, a calcium-dependent transcription factor, in regulating the structural and functional maturation of the hippocampal mossy fiber (MF) synapse.

**Results:**

Using NeuroD2 null mice and *in vivo *lentivirus-mediated gene knockdown, we demonstrate a critical role for NeuroD2 in the formation of CA3 dendritic spines receiving MF inputs. We also use electrophysiological recordings from CA3 neurons while stimulating MF axons to show that NeuroD2 regulates the differentiation of functional properties at the MF synapse. Finally, we find that NeuroD2 regulates PSD95 expression in hippocampal neurons and that PSD95 loss of function *in vivo *reproduces CA3 neuron spine defects observed in NeuroD2 null mice.

**Conclusion:**

These experiments identify NeuroD2 as a key transcription factor that regulates the structural and functional differentiation of MF synapses *in vivo*.

## Background

Excitatory neurotransmission in the central nervous system is mediated by post-synaptic protrusions called dendritic spines [1]. Spines are highly dynamic structures and their growth, stabilization and elimination are proposed to underlie the effects of experience on both the developing and adult brain [2,3]. The effects of neuronal activity on spine morphology are mediated by calcium signaling, which can have acute effects by modulating the existing proteins at the synapse, or can lead to lasting change by transcription-dependent mechanisms. Relatively little is known about how specific transcription factors act to coordinate activity-dependent signaling pathways to influence genes involved in spine morphogenesis.

To identify molecular mediators of activity-dependent development, we previously carried out a screen for calcium-dependent transcription factors expressed in cortical neurons [4]. One gene identified in this screen was the basic helix-loop-helix (bHLH) transcription factor Neurogenic differentiation factor 2 (NeuroD2). Although bHLH genes are best characterized for their role in cell fate determination [5], NeuroD2 is expressed exclusively in post-mitotic neurons [6]. Consistent with a role in activity-dependent development, we found that NeuroD2 regulates thalamocortical connectivity in the mouse somatosensory cortex [7]. Similarly, NeuroD2 has recently been implicated in the differentiation of pre-synaptic terminals using a cerebellar slice co-culture system [8]. These observations motivated us to ask whether NeuroD2 regulates the morphological differentiation of excitatory synapses.

We decided to investigate the role of NeuroD2 in hippocampal synapse formation as hippocampal connectivity is well understood, and distinct classes of synapses can be distinguished using anatomical and functional criteria. One of the most complex synapses in the hippocampus is the mossy fiber synapse, which mediates connectivity between the dentate gyrus (DG) and CA3 regions. This synapse develops entirely during the postnatal period in rodents [9,10]. The post-synaptic specialization of mossy fiber (MF) synapses is characterized by unique multi-headed dendritic spines termed thorny excrescences (TEs), which are engulfed by massive pre-synaptic MF boutons [11,12]. Functionally, MF synapses are characterized by a low probability of release, short-term frequency-dependent facilitation and a unique form of NMDA receptor (NMDAR)-independent, pre-synaptically expressed long-term potentiation [13]. In contrast, distal associational/commissural CA3 synapses form onto classic, mushroom shaped spines, have a higher probability of release and exhibit NMDAR-dependent and post-synaptically expressed long-term potentiation [13].

Here, using NeuroD2 null mice and targeted *in vivo *knockdown of NeuroD2, we investigate the function of this transcription factor on the maturation of the MF synapse. We find that NeuroD2 regulates the elaboration of TE spine heads and the functional differentiation of MF synaptic properties. NeuroD2 also regulates the level of the synaptic scaffolding molecule PSD95 in the developing hippocampus, suggesting that NeuroD2 might influence synaptic structure and function by regulating the expression of scaffolding proteins. Consistent with this possibility, PSD95 loss of function *in vivo *phenocopies the effect of loss of NeuroD2. These results identify NeuroD2 as a key transcriptional regulator of MF connectivity and provide mechanistic insight into the process of MF synapse maturation.

## Results

### Genetic deletion of NeuroD2 leads to a decrease in thorny excrescence spine heads on CA3 neurons

**Figure 1:**
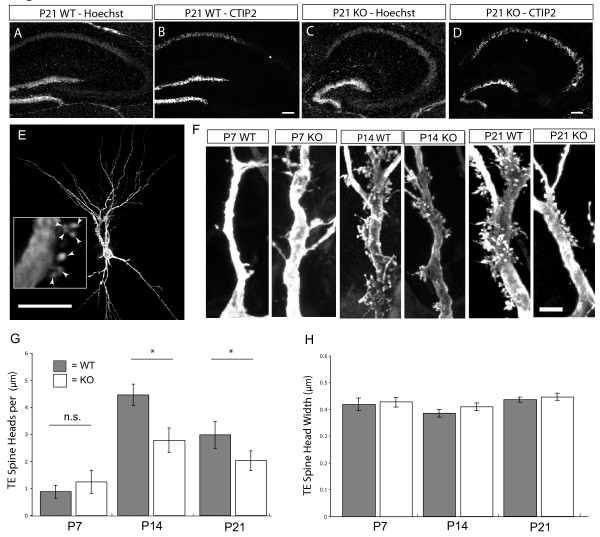
**Genetic deletion of NeuroD2 leads to reduced density of thorny excrescence spine heads**. **(A, C) **Hoechst nuclear staining of the hippocampus from post-embryonic day (P)21 littermate, WT and NeuroD2 null (knockout (KO) **OK**) mice. **(B, D) **CTIP2 immunostaining, specific for DG and CA1 neuron nuclei, shown for the same sections as in (A, C). **(E) **P21 CA3 pyramidal neuron filled in coronal hippocampal section from transcardially perfused mouse by targeted microinjection of Lucifer Yellow dye. Inset indicates individual TE spine heads in a single confocal plane (white arrowheads). **(F) **Representative proximal dendritic segments with TE spines in WT and NeuroD2 null mice (KO) at P7, P14 and P21 time points. **(G) **TE elaboration quantified by counting the density of individual spine heads on primary and secondary dendritic branches in 60X confocal stacks (P7, n = 6 per condition; P14, 21, n ≥ 15). **(H) **Individual TE spine head width quantified in confocal stacks as the width of the head in the confocal plane where it was largest (n ≥ 6 per condition). *P < o.05, *t*-test. Scale bars: 130 μm (A); 75 μm (E); 5 μm (F, I). Error bars represent ± standard error of the mean. N.S., not significant.

NeuroD2 is expressed in the developing cortex and hippocampus during synapse formation [7,14]. To determine if NeuroD2 plays a role in the development of synaptic connectivity in the hippocampus, we analyzed mice in which NeuroD2 has been deleted by homologous recombination [15]. The hippocampus of NeuroD2 null mice is somewhat smaller with a more rounded shape as early as P7, but exhibits normal regional and cellular differentiation based on Hoechst nuclear stain and expression of the DG/CA1 marker CTIP2 (Figure 1A-D). DG axons called mossy fibers extend from DG granule cells and form synapses onto the apical dendrites of CA3 neurons during postnatal hippocampal development. To determine if DG-CA3 synapses were disrupted in the absence of NeuroD2, we examined the morphological development of CA3 dendritic spines.

MF boutons form synapses onto unique dendritic spines, called TEs because of their multi-headed structure with many spine heads emerging from a common neck. An important consideration when examining dynamic structures such as spines is to capture them in their native state. The morphology of some spines is not maintained *in vitro *and spines undergo rapid dynamic responses when sectioned for live cell fills [16]. Thus, to investigate TE formation, we utilized sharp microelectrodes for the current driven injection of Lucifer Yellow (LY) dye into CA3 neurons in fixed tissue slices from transcardially perfused wild-type (WT) and NeuroD2 null mice. Injected dye completely filled dendritic arbors and clearly labeled TE spines on the proximal apical dendrite (Figure 1E, F). CA3 neurons were filled at post-embryonic day (P)7, P14 and P21 in WT and NeuroD2 null littermates (Figure 1F). Multi-headed TE spines cluster onto patches of CA3 dendrites, so to analyze spine density we developed a method to quantify individual TE spine heads using high-resolution image stacks created using confocal microscopy. In WT mice, there was a large increase in TE spine head density between P7 and P14, with a slightly lower density of spine heads at P21 (Figure 1G). NeuroD2 null mice showed no significant difference in TE spine head density compared to controls at P7, but failed to fully elaborate TEs between P7 and P14 when compared with WT littermates, an effect that persisted at P21 (Figure 1G). There were no significant differences in TE spine head width across ages or between conditions (Figure 1H). Thus, the normal elaboration of TE spine heads depends on NeuroD2 function during synapse development.

**Figure 2:**
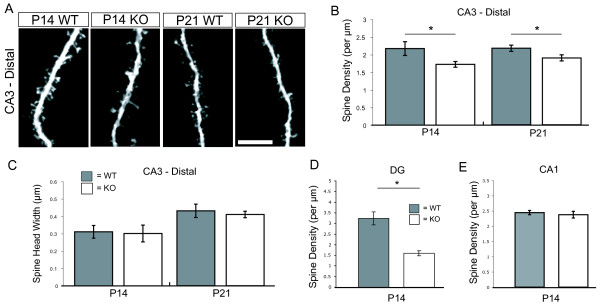
**Genetic deletion of NeuroD2 leads to cell-type-specific effects on classic spine density**. **(A) **Representative CA3 tertiary dendritic segments showing classical spines at P14 and P21. **(B) **Quantification of mean classical spine density on CA3 tertiary segments (n ≥ 9 per condition). **(C) **Quantification of mean CA3 classical spine head width (n ≥ 9 per condition). **(D) **Quantification of DG spine density (WT, n = 7; knockout (KO), n = 8). **(E) **Quantification of CA1 spine density (WT, n = 13; KO, n = 7). *P < 0.05, ***P < 0.001, *t*-test. Scale bar: 5 μm. Error bars represent ± standard error of the mean.

Excitatory synapses onto CA3 neurons in the hippocampus are laminarly distributed such that MF inputs from the DG are formed proximal to the cell body, while CA3-CA3 synapses and entorhinal cortex to CA3 synapses form on the distal dendritic arbor. To determine if the effects of eliminating NeuroD2-mediated transcription were specific to TE elaboration, we examined classical spine density in the same cell fills on distal dendrites, where inputs are largely from other CA3 neurons (Figure 2A). There was a small but significant reduction in distal spine density for both developmental time points, but no effect on distal spine head diameter (Figure 2B, C). These data demonstrate that NeuroD2 also functions to regulate distal spine density, although the effect is not as large as that observed on TE spine elaboration (Figure 1G).

In addition to CA3, NeuroD2 is also highly expressed in both DG and CA1 excitatory neuronal populations. To determine if loss of NeuroD2 resulted in a global decrease in spine density in the hippocampus we also analyzed spine density for these subtypes of hippocampal excitatory neurons. Compared to WT littermates, NeuroD2 null mice exhibited a marked reduction in DG neuron spine density (Figure 2D), but CA1 spine density was unaffected (Figure 2E). These data suggest that NeuroD2 is not required for spine formation in general, but regulates the development of specific classes of synapses.

### NeuroD2 functions cell-autonomously to regulate TE development *in vivo*

**Figure 3:**
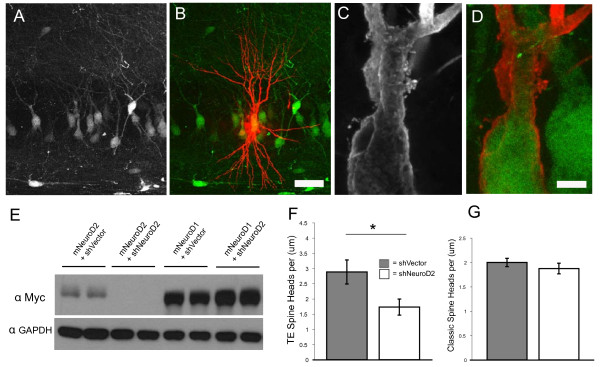
**NeuroD2 functions cell-autonomously to regulate thorny excrescence development *in vivo***. **(A) **Immunohistochemistry (IHC) for lentiviral GFP expression, pups injected at P5 and perfused at P16. **(B) **GFP-positive neuron filled with Lucifer Yellow (LY) dye and processed for IHC against LY (red); GFP-positive neurons are green. **(C) **Higher magnification image of LY IHC showing TE spines. **(D) **Overlaid image of LY and GFP IHC, demonstrating co-localization. **(E) **Immunoblot for myc-tagged NeuroD2 and NeuroD1 co-transfected with either NeuroD2 shRNA expressing lentiviral vector or control vector into 293T cells for 48 hours. Control is blotting for anti-GAPDH for same blot. Loading is duplicate wells. **(F) **Quantification of TE spine head density on primary and secondary dendrites of vector and shNeuroD2 lentivirus-infected neurons filled with LY dye (shVector, n = 14; shNeuroD2, n = 15) **(G) **Quantification of classical spine density on tertiary dendrites of lentivirus-infected neurons filled with LY dye (shVector, n = 15; shNeuroD2, n = 7). *P < 0.05, *t*-test. Scale bar: 50 μm (A, B); 5 μm (C, D). Error bars represent ± standard error of the mean.

Experiments with NeuroD2 null mice do not reveal whether the defect in TE morphology results from a loss of NeuroD2 function in DG or CA3 neurons. To identify the cells in which NeuroD2 function is required, we generated a short hairpin RNA (shRNA) construct against NeuroD2 to cell autonomously reduce NeuroD2 function. This shRNA cassette, when cloned into a lentiviral vector simultaneously expressing green fluorescent protein (GFP), knocked down myc-tagged NeuroD2 expression in 293T cells by greater than 95%, with no effect on NeuroD1, which differs by only four nucleotides in the target region and has the greatest homology with NeuroD2 amongst bHLH transcription factors (Figure 3E). To determine the effect of NeuroD2 knockdown on MF synapse development *in vivo*, we injected lentivirus expressing shNeuroD2 and GFP or GFP alone into the CA3 region of P5 rat pups. CA3 neurons were clearly labeled with lentiviral GFP expression in slices cut from transcardially-perfused rats at P16 (Figure 3A). To accurately quantify spine morphology, we developed a technique for targeted microinjection of LY dye into GFP labeled neurons guided by fluorescence microscopy. The LY signal was amplified with an anti-LY antibody (Figure 3B) and the filled neuron was confirmed *post hoc *by immunostaining for GFP (Figure 3B-D). TE spines were clearly visible on CA3 neurons filled in this manner (Figure 3C). *In vivo *shRNA-mediated knockdown of NeuroD2 reduced the density of TE spine heads approximately 40%, similar to the effect observed in NeuroD2 null mice, indicating that NeuroD2 can cell-autonomously regulate the structural elaboration of TE spine heads (Figure 3F). Interestingly, quantification of distal classical spines did not show a significant reduction between control and NeuroD2 shRNA virus (Figure 3G). These data suggest that either NeuroD2 regulates distal spine density in a non-cell autonomous manner or that NeuroD2 function is critically and specifically involved in TE elaboration during the time window of our acute manipulation.

### Functional maturation of mossy fiber synaptic properties is impaired in NeuroD2 nulls

**Figure 4:**
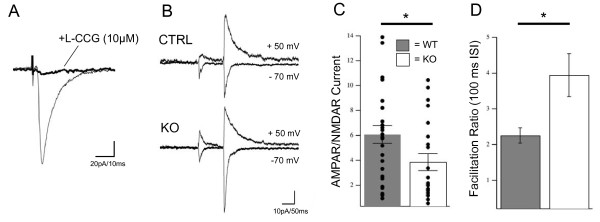
**Functional maturation of mossy fiber synaptic properties is impaired in NeuroD2 null mice**. **(A) **Representative whole cell voltage-clamp recording in an acute sagittal slice from P14 to P16 mouse while stimulating MF axons. A representative evoked synaptic current is shown at a holding potential of -70 mV, before and after perfusion with L-CCG (10 μM). **(B) **Evoked MF responses demonstrating strong paired-pulse facilitation and showing currents recorded at holding potentials of +50 and -70 mV. **(C) **Quantification of AMPA receptor (AMPAR)/NMDAR-mediated current ratios at the MF synapse, measured as the peak current at -70 mV relative to the peak current 50 ms into the response at +50 mV (WT = 6.08 ± 0.70, n = 27; knockout (KO) = 3.86 ± 0.68, n = 22). **(D) **Quantification of facilitation ratios measured as peak current of second response relative to first response at a holding potential of -70 mV and a 100 ms inter-stimulus interval (ISI; WT = 2.26 ± 0.21, n = 9; KO = 3.95 ± 0.60, n = 11). *P < 0.05, *t*-test. Error bars represent ± standard error of the mean.

To examine the effect of loss of NeuroD2 on MF functional properties, we carried out voltage-clamp recordings from CA3 neurons while stimulating MF axons in acute brain slices from WT and NeuroD2 null mice. Current responses to MF stimulation showed short latencies, rapid rise times and underwent strong paired-pulse facilitation (PPF), all of which are key features of monosynaptic responses at this synapse [17] (Figure 4A, B). Additionally, MF-evoked synaptic responses were strongly suppressed by perfusion with L-CCG (10 μM), a group II metabotropic glutamate receptor (mGluR) agonist that acts on mGluRs expressed specifically at MF terminals to suppress neurotransmitter release and has been used in a number of studies to confirm MF synapse identity (Figure 4A) [18-23]. The amplitude and response kinetics of CA3 neurons elicited by MF stimulation were similar between WT and NeuroD2 null conditions (data not shown).

The postnatal maturation of glutamatergic synapses is marked by a large increase in the ratio of current carried by AMPA receptors (AMPARs) in relation to NMDARs (AMPAR/NMDAR ratio) [24]. This ratio is therefore commonly used to measure the state of glutamatergic synapse maturation. We measured AMPAR/NMDAR-mediated current ratios at the MF synapse in WT and NeuroD2 null brain slices. To determine the AMPAR-mediated current we recorded the peak inward current evoked by MF stimulation at -70 mV, at which level the NMDAR-mediated current is blocked by magnesium ions. The AMPAR-mediated current was then divided by the current amplitude measured 50 ms post-stimulus and at a holding potential of +50 mV, where current is largely mediated by NMDARs. The AMPAR/NMDAR ratio was significantly reduced in NeuroD2 null mice compared to littermate controls, suggesting that the proper functional maturation of the DG-CA3 synapse requires NeuroD2 (Figure 4C).

A characteristic feature of the MF to CA3 synapse is a strong PPF, which is a measure of presynaptic function and scales inversely with the probability of release at a synapse. In order to determine if PPF was affected in NeuroD2 null mice we delivered two stimuli to the MF pathway with an interstimulus interval set at 100 ms. The facilitation ratio was calculated at a holding potential of -70 mV by taking the peak amplitude of the second excitatory postsynaptic current and dividing by the peak of the first excitatory postsynaptic current (Figure 4B). The PPF ratio was nearly doubled for NeuroD2 null MF synapses when compared to controls (Figure 4D), suggesting that MF synapses in NeuroD2 null mice exhibit a significantly lower probability of release than WT synapses. These observations further support an important role of this transcription factor in the proper developmental maturation of the MF to CA3 synapse.

### NeuroD2 regulates MF-CA3 synaptic connectivity *in vitro*

**Figure 5:**
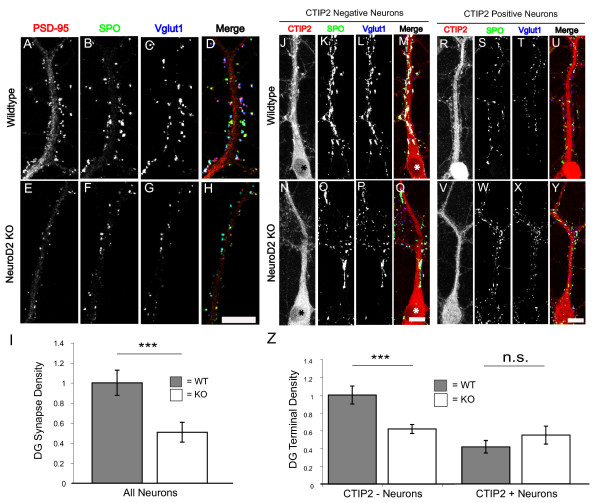
**DG-CA3 synapses are reduced in NeuroD2 null neurons *in vitro***. Hippocampal neurons from P0 WT and NeuroD2 null mice were cultured at low density on a glial monolayer and fixed for immunostaining at 16 days *in vitro *for the following markers: **(A, E) **PSD-95; **(B, F) **synaptoporin (SPO); **(C, G) **Vglut1; **(D, H) **merged images of staining for synaptically localized proteins with PSD-95 in red, SPO in green and Vglut1 in blue. **(I) **Quantification of DG synapse density by triple co-localization of synaptic markers on large primary dendrites (WT, n = 25; knockout (KO), n = 21). Cultures were also immunostained with the DG- and CA1-specific nuclear marker CTIP2 to investigate the localization of synaptic terminals onto putative hippocampal CA3 neurons *in vitro*. **(J, M, N, Q) **CTIP2-negative neurons, putative CA3 neurons, lacked staining in their nucleus; the asterisk indicates lack of nuclear staining for CTIP2. **(R, U, V, Y) **CTIP2-positive neurons, non-CA3 neurons, had staining that included the nucleus. **(K-M, O-Q, S-U, W-Y) **SPO and Vglut1 immunohistochemistry were used to localize MF terminals onto the proximal dendrites of these neuron types as indicated. **(Z) **Quantification of DG terminal density onto CTIP2-negative, putative CA3 neurons (WT, n = 14; KO, n = 16), and CTIP2-positive non-CA3 neurons (WT, n = 9; KO, n = 8). ***P < 0.01, *t*-test; n.s., not significant. Scale bars: 10 μm. Error bars represent ± standard error of the mean. (Note that the secondary antibody used to detect CTIP2 cross-reacts with an additional primary antibody against CamKII used as a dendritic marker, leading to dendritic signal in the red channel that did not interfere with assessing nuclear CTIP2 signal).

To determine how loss of NeuroD2 affects the localization of synaptic proteins, we next analyzed hippocampal neuronal cultures from WT and NeuroD2 null littermates. MF synapses *in vitro *were defined as the co-localization of the MF specific pre-synaptic marker synaptoporin (SPO), the excitatory pre-synaptic marker vesicular glutamate transporter 1 (Vglut1) and the post-synaptic scaffolding molecule PSD95 (Figure 5A-H) [25,26]. Consistent with the morphological analysis *in vivo*, there was a highly significant reduction in the density of MF synapses on the large proximal dendrites of pyramidal shaped neurons in NeuroD2 null cultures compared to controls (Figure 5I).

To specifically address the role of NeuroD2 in regulating MF synapse density on CA3 neurons, we combined staining for the pre-synaptic markers SPO and Vglut1 with the cell-type specific marker CTIP2. CTIP2 is a transcription factor that is highly expressed in dentate granule neurons and CA1 pyramidal neurons, but is completely absent from CA3 pyramidal neurons (Figure 1C, D); therefore, CTIP2-negative cells are presumptive CA3 neurons [26,27]. Immunohistochemistry for CTIP2 *in vitro *reveals two clear populations of neurons, putative CA1 and DG neurons with CTIP2 staining that includes the nucleus (Figure 5R, V), and putative CA3 neurons, which completely lack CTIP2 nuclear staining (Figure 5J, N). We analyzed SPO and Vglut1 co-localization on these two classes of neurons to assess the density of DG pre-synaptic terminals onto these postsynaptic populations (Figure 5J-Y). Consistent with a CA3 neuron identity, CTIP2-negative neurons in culture received greater numbers of MF terminals onto their proximal dendrites when compared to CTIP2-positive neurons (Figure 5Z). CTIP2-negative neurons (presumptive CA3 neurons) from NeuroD2 null cultures showed an approximately 40% reduction in DG terminals, while there was no difference in DG terminals onto incorrect target cells that were CTIP2 positive (Figure 5Z). These data are consistent with our finding of decreased TE density *in vivo *and indicate that NeuroD2 is specifically required for the formation of DG terminals onto CA3 neurons.

### Regulation of synaptic scaffolding proteins by NeuroD2

**Figure 6:**
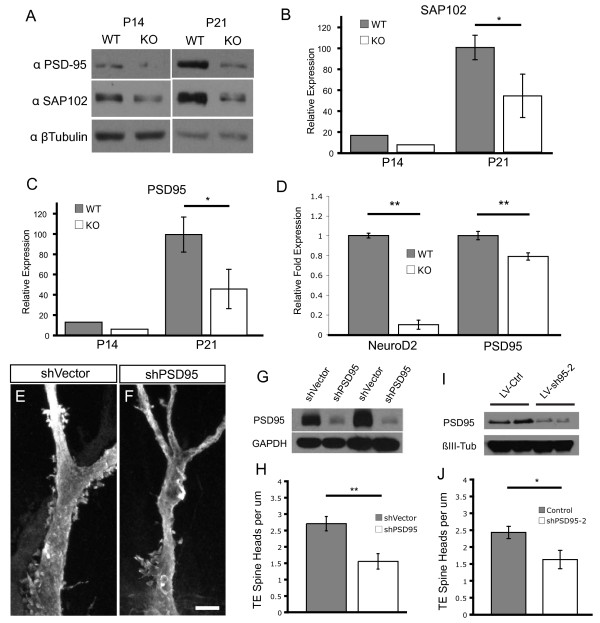
**NeuroD2 regulates expression of PSD95 and SAP102 in the developing hippocampus**. **(A) **Representative immunoblots against PSD95 and SAP102 in hippocampal lysates from WT and NeuroD2 null littermates at P14 and P21. Loading control is immunoblotting for β-tubulin. **(B) **Quantification of SAP102 relative expression (n = 3 littermate pairs). **(C) **Quantification of PSD95 relative expression as in (B) (n = 3 littermate pairs). **(D) **Quantitative PCR analysis of NeuroD2 and PSD95 mRNA expression in WT and NeuroD2 null cultures (n = 5 and n = 6, respectively). **(E) **LY filled, shVector-infected CA3 neuron. **(F) **LY filled, shPSD95-infected CA3 neuron. **(G) **Immunoblot for mCherry-tagged PSD95, co-transfected with PSD95 shRNA expressing lentiviral vector or control vector into 293T cells for 48 hours (duplicate experiment). Control is blotting with anti-GAPDH. **(H) **Quantification of TE spine head density between conditions (shVector, n = 9; shPSD95, n = 8). **(I) **Immunoblot for endogenous PSD95 in neuronal culture infected with a lentivirus expressing a second shPSD95 construct. **(J) **Quantification of TE spine head density with an independent construct, shPSD95-2 (control, n = 12; shPSD95-2, n = 15). *P < 0.05, **P < 0.01. Scale bar: 5 μm. Error bars represent ± standard error of the mean. KO, knockout.

Transcription factors influence the structure and function of neurons via their regulation of specific sets of genes within transcriptional networks. To explore the possibility that NeuroD2 regulates the expression of synaptic proteins, we examined the levels of synaptic scaffolding molecules known to regulate the structure and function of synapses. Using western blot analysis with hippocampal lysates we found that the levels of the membrane-associated guanylate kinase synaptic scaffolding molecules SAP102 and PSD95 were reduced in NeuroD2 null mice compared to littermates at both P14 and P21 by greater than 50% (Figure 6A-C). Similarly, relative expression of PSD95 mRNA was significantly reduced in NeuroD2 null neurons *in vitro *compared to WT controls by quantitative PCR analysis (Figure 6D). Importantly, several other synaptic proteins were not reduced in the NeuroD2 null hippocampus based on immunoblot analysis (Additional file 1). These observations indicate that expression of two key scaffolding proteins, PSD95 and SAP102, are strongly influenced by NeuroD2 levels and suggest that NeuroD2 may exert its effects on MF-CA3 synapse maturation via regulation of these molecules.

To determine if the reduction in PSD95 levels in NeuroD2 null mice might account for the observed TE spine defect, we examined the effect of PSD95 loss of function using lentiviral expression of an shRNA construct (shPSD95). The PSD95 shRNA and lentiviral vector we used for these experiments have been extensively analyzed to confirm both efficacy and specificity [28], and we confirmed that this PSD95 shRNA led to significant knockdown of PSD95 expressed in 293T cells (Figure 6G). To assess the role of PSD95 on TE development *in vivo*, we did targeted injections of lentivirus expressing shPSD95 and GFP or GFP alone into the CA3 region of P5 rat pups. Knocking down PSD95 expression in the developing rat hippocampus, from P5 to P16, was sufficient to reduce elaboration of TE spines by nearly 50% (Figure 6E-G), which is similar to the reduction seen in NeuroD2 null mice. To confirm this effect, we developed a second shRNA-expressing construct targeted against an independent region of the PSD95 gene. In addition to knockdown of PSD95 exogenously expressed in 293T cells, we further confirmed knockdown of endogenous protein in cultured hippocampal neurons for this construct (Figure 6I). For these experiments we utilized *in utero *co-electroporation of the shRNA-expressing construct with a construct expressing membrane bound GFP compared with constructs expressing GFP alone. This experiment also demonstrated an shRNA-dependent decrease in the density of TE spine heads of similar magnitude, confirming the role of PSD95 in the development of these post-synaptic structures (Figure 6J). These experiments demonstrate that PSD95 expression is reduced in the absence of NeuroD2 and that PSD95 is critical for the normal development of TE spines.

## Discussion

**Figure 7:**
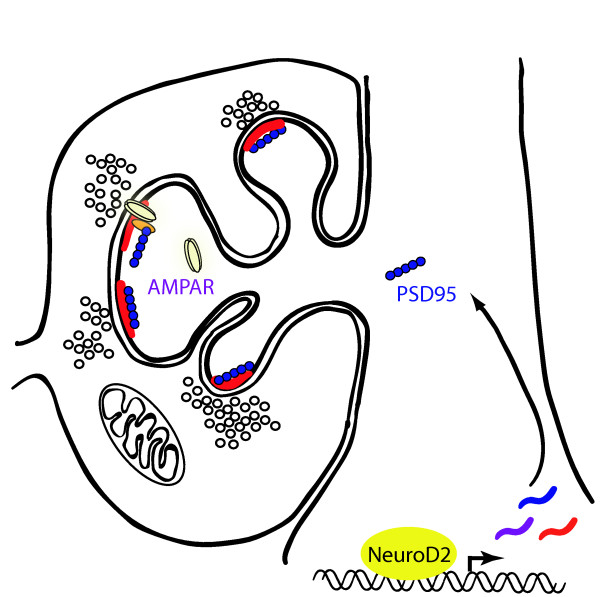
**Diagrammatic representation of the proposed role of NeuroD2 in thorny excrescence spine maturation**. NeuroD2 is a calcium-activated transcription factor that regulates the transcription of the post-synaptic scaffolding molecule PSD95 amongst other target genes. PSD95 expression downstream of NeuroD2 is part of a pathway regulating the elaboration of TE spine heads during MF synapse development. NeuroD2 also regulates the functional maturation of glutamatergic currents at the MF synapse, an effect that may relate to the role of PSD95 in modulating the AMPAR content of developing synapses.

The observations reported here identify NeuroD2 as a key transcriptional regulator of MF synapse development. In NeuroD2 null mice there is a marked decrease in the density of TE spine heads on the proximal apical dendrites of CA3 neurons. Lentiviral-mediated shRNA knockdown of NeuroD2 *in vivo *further demonstrates that NeuroD2 functions cell-autonomously to regulate the elaboration of TE spine heads. Further, the similarity in spine phenotypes resulting from loss of NeuroD2 and PSD95, together with the observation that PSD95 levels are significantly reduced in NeuroD2 nulls, suggests that NeuroD2 exerts its effects on synapse differentiation at least in part via regulation of the synaptic scaffolding protein PSD95 (Figure 7).

Reduced TE elaboration in NeuroD2 nulls could reflect either a decreased density of MF synapses or a less mature state of synaptic differentiation, possibilities that are not mutually exclusive. Hippocampal cultures from NeuroD2 null mice show a large reduction in the density of MF synapses as defined by the co-localization of pre- and post-synaptic molecules. This experiment distinguishes the NeuroD2 null phenotype from an axon guidance defect as guidance mechanisms likely do not function in dissociated cultures. The use of cell-type- and synapse-specific markers demonstrates that NeuroD2 regulates the density of MF terminals on the proximal dendrites of presumptive CA3 neurons *in vitro *with no effect on MF terminal density onto incorrect targets. These observations identify NeuroD2 as a critical regulator of DG-CA3 synapses and suggest that this transcription factor is responsible for the differentiation of MF-specific synaptic features.

Our electrophysiological recordings demonstrate that the functional maturation of MF synaptic properties is also disrupted in the absence of NeuroD2. The ratio of AMPAR/NMDAR-mediated currents increases during development [24,29-31]. Recordings made from CA3 neurons while stimulating MF inputs demonstrate a reduction of AMPAR/NMDAR-mediated current ratio in NeuroD2 null mice. NeuroD2 null mice also exhibit an increased PPF ratio, indicating that NeuroD2 can also likely function to regulate release probability at the pre-synaptic terminal. These results point to a critical role for NeuroD2 in the functional maturation of individual MF-CA3 synapses.

That NeuroD2 regulates the expression of post-synaptic proteins such as PSD95 and SAP102 indicates that these molecules may mediate the effects of NeuroD2 on synaptic differentiation. PSD95 overexpression *in vitro *drives increased spine density and head size [32]. However, loss of function experiments *in vitro *have yielded mixed results on spine morphogenesis ([28] versus [33]). Prior to this work, the role of PSD95 on *in vivo *spine morphology has not been extensively studied, with most studies done *in vitro *[32,33]. Similarly, very little is known about the function of these molecules specifically at the MF synapse. In our experiments, *in vivo *knockdown of PSD95 in CA3 neurons leads to a cell-autonomous reduction in the developmental elaboration of TE spines, mimicking the effect of NeuroD2 loss of function experiments. These data indicate that reduced expression of PSD95 downstream of NeuroD2-regulated transcription is likely to directly influence post-synaptic morphology at the MF synapse *in vivo*.

PSD95 and SAP102 have also been demonstrated to regulate the AMPAR content of developing synapses [34], and may therefore underlie the reduced AMPAR/NMDAR-mediated current ratio at MF synapses in the NeuroD2 null hippocampus. SAP102 is expressed prior to PSD95 and accumulates first at hippocampal synapses *in vivo *[35,36]. Each molecule has differential effects during synaptogenesis and synapse maturation. Knockdown of SAP102 transiently impairs glutamatergic currents at P7 CA3-CA1 synapses *in vivo*, while knockdown of PSD95 has no effect at P7, but dramatically suppresses AMPAR accumulation at P16 [37]. Additional evidence from a number of studies indicates synapse specific and developmentally regulated functions of membrane-associated guanylate kinase scaffolds in glutamatergic phenotype [28,34,38]. Coordinated regulation of scaffolding molecules by NeuroD2 may reflect a general mechanism by which transcription factors influence synaptic differentiation and maturation.

It is noteworthy that NeuroD2 appears to have both cell-type- and synapse-specific effects. Our result indicate that, in the absence of NeuroD2-mediated transcription, DG and CA3 neuron spine density is decreased while CA1 neuron spine density appears to be intact. Interestingly, the most extensive study of spine development in PSD95 null mice found no overall effect on CA1 spine density, but a significant reduction in spine density of entorhinal cortex neurons [39]. Given that PSD95 is widely expressed in the hippocampus and localizes to multiple synapse types, how might decreased PSD95 expression downstream of NeuroD2 mediate such specific effects? One intriguing possibility is that PSD95 might act to bring together functional complexes with unique constituents depending on synapse type or cellular context, such that its loss of function yields differential effects. This suggests that synapse-specific analysis of PSD95 interacting proteins may be fruitful in identifying molecules that mediate synaptic specificity.

Our experiments exploring the effects of reduced PSD95 expression in the hippocampus of NeuroD2 null mice do not rule out the possibility that additional molecules might also play a role in aspects of the synaptic phenotype. For example, expression of the cell adhesion molecules SynCAM1 to -3 are developmentally regulated by NeuroD2, and levels of SynCAM3 are strongly reduced in the NeuroD2 null hippocampus at both P14 and P21 (Additional file 2). Our analysis of SynCAM1 and SynCAM3 null mice did not reveal a decrease in TE spines (data not shown); however, these molecules may contribute to other aspects of the NeuroD2 null phenotype.

Transcriptional regulation of synaptic connectivity has recently been a topic of considerable interest. Greenberg and colleagues [40-42] have demonstrated that specific activity-regulated transcription factors are involved in synapse elimination and inhibitory synapse formation. More recently, Bonni and colleagues [8] have specifically implicated NeuroD2 in a pathway regulating the differentiation of pre-synaptic terminals in the cerebellum. Our study further indicates a central role for NeuroD2 in the transcriptional regulation of synapse differentiation. That NeuroD2 regulates the expression of post-synaptic proteins in the hippocampus suggests that decreased levels of these molecules might underlie the structural and functional deficits observed in NeuroD2 null mice. NeuroD2 and other bHLH transcription factors regulate gene expression by binding E-Box sequences (CANNTG) upstream of transcriptional start sites [43]. Both PSD95 and SAP102 have a number of potential E-Box binding sites, which may act as NeuroD2 regulatory elements (Additional file 3). Further, PSD95 expression is regulated by activity in the mouse cochlea and is necessary for the *in vitro *maturation of hippocampal synapses downstream of neuronal activity [33,44]. The hippocampal MF synapse develops entirely during the postnatal period and thus during a period when an animal is exposed to a diverse set of environmental influences [9,10]. The long-term effects of such activity at the neuronal level are determined by calcium-regulated transcription [42]. As a calcium-dependent transcription factor, NeuroD2 may function as part of a regulatory pathway matching PSD95 expression to the level of synaptic activity seen by a particular neuron and thereby contribute to activity-dependent synaptic development.

## Conclusion

Here we demonstrate a critical role for the transcription factor NeuroD2 in the development of the hippocampal MF synapse. We show PSD95 expression is reduced in the absence of NeuroD2 and that this decrease pheno-copies the effect of NeuroD2 loss of function on TE development at the MF synapse. These observations advance our understanding of the transcriptional control of synaptic differentiation. Together with other studies this work begins to provide a framework for how activity-dependent transcription factors can shape the organization of neural circuits.

## Materials and methods

All experiments have been carried out under the University of California, San Diego's Animal Care and Use Committee guidelines.

### Plasmids

For shRNA-mediated knockdown, candidate shRNA sequences were purchased from Open Biosystems (Lafayette, CO, USA) in the pLKO.1 expression vector driving shRNA expression off the U6 promoter and evaluated for efficacy and specificity. For shRNA-mediated knockdown of NeuroD2, we used the following sequence corresponding to nucleotides 911 to 931 of rat NeuroD2: GCTCTGTCTCAACGGCAACTT. Mouse and rat NeuroD2 are 100% conserved in the target region for the shRNA. For *in vivo *knockdown of NeuroD2 expression, the U6 promoter and shNeuroD2 sequence from PLKO.1 were cloned into the PacI site of the lentiviral vector plasmid FCK(0.4)GW (a gift from Dr Pavel Osten, Cold Spring Harbor Laboratory, Cold Spring Harbor, NY), which contains a 0.4 kb fragment of the mouse CaMKII promoter driving EGFP [45]. As a control vector, we used FCK(0.4)GW alone. Myc-tagged mouse NeuroD2 and NeuroD1 were expressed from the pCS2+ plasmid off the CMV promoter. Validated lentiviral constructs expressing shRNA against PSD95 and empty vector control expressing GFP alone were obtained in the pLLox3.7 vector (a gift from Dr Roger Nicoll, UCSF, San Francisco, CA). The shRNA sequence for PSD95 was: TCACGATCATCGCTCAGTATA. These constructs have been extensively validated for efficacy and specificity in previous publications [28]. A second independent PSD95 shRNA and GFP expressing lentiviral construct in the pGIPZ vector (Open Biosystems) was used for confirmation, for which the sequence was CAGCACATCCCTGGAGATA. This second construct or a control construct (FCK(0.4)GFP) was combined with pFCK(0.4)mGFP, expressing membrane-targeted GFP for *in utero *electroporation experiments.

### Intracellular injection of Lucifer Yellow

Mice of either sex were anesthetized and transcardially perfused with phosphate-buffered saline (PBS) followed by 4% paraformaldehyde at pH 7.4 and post-fixed in 4% paraformaldehyde for 1 hour on ice. Then, 100 μm thick coronal sections were cut using a vibratome and stored in PBS on ice. Penetrating microelectrodes were pulled from standard borosilicate capillary glass with filament (1 mm outer diameter/0.58 mm inner diameter) and back-filled with LY dye (5%). Slices were mounted on coverslips under PBS and CA3 neurons were filled via iontophoresis under visual guidance. Sections were then post-fixed an additional 15 minutes before being prepared for immunohistochemistry.

### *In utero *electroporation

Timed-pregnant CD-1 white mice (Charles River, E15) were anesthetized with 3% isoflurane. A small vertical incision was made in the skin and abdominal wall and embryos gently exposed. Each embryo was injected with 1 to 2 μl of DNA solution and 0.01% Fast Green into the ventricles [46]. For spine analysis, pGIPZ-shPSD95 plasmid DNA or pFCK(0.4) was mixed with pFCK(0.4)-mGFP DNA. A pressure-controlled beveled glass pipette (Drummond (Broomall, PA, USA), WPI Microbeveller (Sarasota, FL, USA)) was used for injection. After each injection, the embryos were moistened with PBS and voltage steps via tweezertrodes (BTX, 5 mm round, platinum, BTX electroporator (Holliston, MA, USA)) were applied at a 30 to 45 degree angle with respect to the interaural line to target CA3. Voltage was 36V for 5 pulses at 1 Hz, each pulse lasting 50 ms.

### Imaging and analysis of spines

Spines were imaged on a Leica SP2 or SP5 confocal microscope under 63 × magnification with 3 × optical zoom for imaging of TE spines and 4 × optical zoom for imaging of classical spines. Images were collected from clearly identifiable pyramidal neurons throughout the extent of CA3 with TE spine images collected on primary and secondary dendrites and classic spine images collected on tertiary branches in the middle third of the CA3 stratum radiatum. Images were collected as z-stacks with 0.5 μm thick sections. Images were analyzed as confocal stacks using ImageJ software. For all spines, individual spine heads were identified and analyzed in the confocal plane in which they had the largest area. Head width measurements were obtained using a custom ImageJ plugin called edgefitter [47]. Individual multi-headed spines cannot be separated at the light level, but individual spine heads could be reliably separated using high-resolution confocal stacks and our analysis is of overall TE spine head density.

### Lentivirus production

Lentivirus was made by transfecting 293T cells with the pFCK(0.4)GW plasmid along with helper plasmids (psPAX2 and VSVG). After 3 days, 293T media was centrifuged (46,000 × g) to concentrate the virus, resuspended in PBS, and stored at -80°C. For *in vivo *injections, P5 rat pups of either sex were anesthetized using an isoflurane vaporizer and immobilized in a stereotaxic device. Following craniotomy, a Hamilton syringe was used to inject 1 μl of concentrated virus. The animals were sutured and returned to their cage until further analysis.

### Electrophysiology

Whole-cell, voltage-clamp recordings were performed on CA3 pyramidal neurons in acute brain slices from NeuroD2 null mice and littermate controls. Mice of either sex aged P14 to P17 were deeply anesthetized with isoflurane and then rapidly decapitated. Brain slices were cut in the sagittal plane at a thickness of 350 μm using a vibrating microtome (VT-1200, Leica Microsystems, Bannockburn, IL, USA). Slicing was performed in an ice-cold artificial cerebral spinal fluid with the following ionic composition (concentrations in mM): NaCl 124, KCl 5, NaHCO_3 _26, NaH_2_PO_4 _1.25, glucose 10, MgCl_2 _6, CaCl_2 _1. Slices were moved directly to a holding chamber and maintained for at least 0.5 hours (0.5 to 3 hours), and then transferred to a recording chamber. In both the holding and recording chambers, slices were submerged in a standard artificial cerebral spinal fluid solution (at room temperature) with the following ionic composition (in mM): NaCl 124, KCl 5, NaHCO_3 _26, NaH_2_PO_4 _1.25, glucose 10, MgCl_2 _3, CaCl_2 _2, and bubbled constantly with 95% O2:5% CO_2 _gas. Our recording solution also contained 100 μM picrotoxin (Tocris Bioscience (Minneapolis, MN, USA)) to block GABA receptor-mediated inhibitory currents. CA3 pyramidal neurons were visualized by infrared differential interference contrast imaging using an upright, fixed-stage microscope (BX-51, Olympus (Center Valley, PA, USA)). Whole-cell patch electrodes were pulled from borosilicate glass (1.5 mm outer diameter and 1.16 mm inner diameter, Warner Instruments (Hamden, CT, USA)) to resistances ranging from 3 to 6 MΩ. Access resistance was monitored for consistency during recordings and ranged from 10 to 20 MΩ for the cells included for analysis. The intracellular recording solution contained (in mM): CsCl 20, CsMeSO_3 _105, ATP (dipotassium salt) 0.5, GTP 0.3, Hepes 10, MgCl_2 _2, EGTA 1, QX-314 2 and BAPTA 10, at pH 7.3. Recordings were acquired using a PC-505 amplifier (Warner Instruments (Hamden, CT, USA)) and digitized using custom software routines written in Igor Pro (Wavemetrics (Portland, OR, USA)) and the NIDAQ tools package, to access a PCI-based board (PCI-1200, National Instruments (Austin, TX, USA)) on a Macintosh G3 computer.

### Hippocampal culture

Hippocampal neurons were cultured from P0 WT and NeuroD2 null mouse littermates of either sex. The whole hippocampus, including DG, CA3 and CA1 regions, was dissected free from the cortex, and neurons were dissociated and plated on a rat glial monolayer previously cultured on poly-D-lysine (Millipore, Temecula, CA, USA) and laminin (Invitrogen, Carlsbad, CA, USA) coated coverslips. Neurons were maintained in Neurobasal-A medium (Invitrogen) supplemented with B27, glucose, glutamax, penicillin/streptomycin (Invitrogen) and 25 μM B-mercaptoethanol.

### Immunohistochemistry

For LY immunohistochemistry, slices were blocked 1 hour at room temperature in 3% bovine serum albumin + 0.3% Triton X-100 in PBS and incubated in primary antibody against LY (Abcam, rabbit anti-LY (Cambridge, MA, USA); 1:1,000) overnight at 4°C in blocking solution. Slices were then washed three times for 20 minutes each with blocking solution and incubated in secondary antibody (Molecular Probes, donkey anti-rabbit 555 (Grand Island, NY, USA); 1:1,000) for 2 hours at room temperature. Finally, slices were labeled with a Hoechst nuclear stain and mounted on slides for confocal microscopy. For virally labeled neurons, slices were simultaneously incubated with primary antibody against GFP (Abcam, goat anti-GFP; 1:3,000) and stained with a secondary antibody in the far red channel (Molecular Probes, donkey anti-goat 647, 1:1,000).

For immunohistochemistry in slices from WT and NeuroD2 null mice, animals of either sex were transcardially perfused as for microinjection of LY, but were post-fixed overnight at 4°C. Slices for immunohistochemistry were saturated with a 30% sucrose in PBS solution for approximately 3 days and flash frozen in dry ice before cutting 30 μm frozen sections on a cryostat microtome. Slices were then blocked with 3% bovine serum albumin in PBS for 1 hour before being incubated in primary antibody overnight at 4°C, washed and incubated with fluorophore conjugated secondary antibody for 2 hours at room temperature, and washed and incubated with Hoechst nuclear stain before being coverslipped.

Cultured neurons were fixed in 4% paraformaldehyde, 4% sucrose in PBS and processed for immunofluorescence according to standard procedures. Cells were washed with PBS and incubated in blocking solution (PBS plus 3% bovine albumin and 0.1% TritonX 100) for 30 minutes. For SPO staining only, 0.1% Saponin was included only in the preliminary blocking step. Then cells were incubated in primary antibody diluted in blocking solution for 2 hours, washed, incubated in secondary antibody diluted in blocking solution for 1 hour, washed again and stained with Hoechst nuclear stain before being coverslipped. Primary antibodies were: rat anti-CTIP2 1:1,000 (Abcam), rabbit anti-synaptoporin 1:1,000 (Synaptic Systems, (Goettingen, Germany)), mouse anti-PSD95 1:250 (NeuroMAB, (Davis, CA, USA)), guinea-pig anti-Vglut1 1:5,000 (Chemicon, (Billerica, MA, USA)), Chicken anti-MAP2 1:5,000 (Abcam). Fluorophore-conjugated secondary antibodies were from Jackson ImmunoResearch (West Grove, PA, USA) or Invitrogen and were used at 1:1,000.

### Image acquisition and analysis of *in vitro *experiments

Images were captured on Leica SP2 and SP5 confocal microscopes (Leica Microsystems (Buffalo Grove, IL, USA)). Z-Stacks were collapsed in a maximum projection and analyzed using NIH ImageJ software. Images were thresholdeded using constant settings per experiment and colocalized synaptic markers were quantified per unit length of dendrite using an automated method of assessing pixel overlap.

### Immunoblotting and quantification

Western blotting was performed by standard procedures. For hippocampal lysates, mice of either sex were decapitated under isoflurane anesthesia, and the hippocampus was dissected free in cold PBS and homogenized in buffer with protease inhibitors. For 293T expression experiments cells were lysed directly in hot SDS containing sample buffer and boiled for 10 minutes. Lysates were diluted into SDS containing sample buffer, boiled for 10 minutes and run by SDS-PAGE. Antibodies used for immunoblotting were the following: mouse anti-myc, 1:1,000 (Santa Cruz Biotechnology (Santa Cruz, CA, USA)), mouse anti-PSD95 1:1,000 (Affinity Bioreagents ABR (Rockford, IL, USA)), mouse anti-βIIItubulin 1:1,000 (Abcam), mouse anti-GAPDH 1:5,000 (Millipore (Billerica, MA, USA)), mouse anti-Pick1 1:500 (NeuroMAB), mouse anti-SAP102 1:1,000 (NeuroMAB), rabbit anti-GluR2 1:1,000 (Chemicon), mouse anti-NR2A 1:1,000 (Chemicon), mouse anti-NR2B 1:1,000 (NeuroMAB), rat anti-synaptoporin 1:1,000 (Synaptic Systems), rabbit anti-CKII 1:1,000 (Upstate Biotechnology (Billerica, MA, USA)), rabbit anti-BDNF 1:1,000 (Santa Cruz Biotechnology). Immunoblotting and quantification for SynCAM1 to -3 were performed as previously described [48]. Quantification of other immunoblots was by measurement of densitometry in standard sized windows, using imageJ software.

### Quantitative PCR experiments

Mouse cortical neurons were taken from embryonic 15 day pups of either sex, cultured in Neural Basal Medium with B27 and stimulated with indicated methods at 3 and 12 days *in vitro*, respectively. Total RNA was collected 3 hours after stimulation and reverse-transcribed to cDNA. Real-time PCR are performed with SYBR-Green dye on Rotor-Gene Q (Qiagen (Valencia, CA, USA)). Statistical analysis is done with software with Rotor-Gene.

## Abbreviations

AMPAR: α-amino-3-hydroxy-5-methyl-4-isoxazolepropionic acid receptor; bHLH: basic helix-loop-helix; DG: dentate gyrus; GFP: green fluorescent protein; LY: Lucifer Yellow; MF: mossy fiber; mGluR: metabotropic glutamate receptor; NeuroD2: Neurogenic differentiation factor 2; NMDAR: N-methyl D-aspartate receptor; P: post-embryonic day; PBS: phosphate-buffered saline; PPF: paired-pulse facilitation; shRNA: short hairpin RNA; SPO: synaptoporin; TE: thorny excrescence; Vglut1: vesicular glutamate transporter 1; WT: wild type.

## Competing interests

The authors declare that they have no competing interests.

## Authors' contributions

SAW was involved in performing and analyzing all experiments except electrophysiology and quantitative PCR. BJH performed and analyzed electrophysiology. JKA assisted with imaging and spine analysis. LAD performed *in utero *electroporation and imaging. BJH, FC, SO, MEW, ZQ and BY assisted with molecular biology and performed quantitative PCR experiments. EMR performed and analyzed immunoblots for SynCAM1 to -3. KT assisted with lentivirus injections. AG and TB participated in the design of experiments. All authors participated in the preparation of the manuscript. All authors read and approved the final manuscript.

## Supplementary Material

Additional file 1 **Some synaptic proteins are unaffected in the NeuroD2 null hippocampus**. **(A) **Representative immunoblots against hippocampal lysates from P21 WT and NeuroD2 null littermates. **(B) **Quantification of immunoblots against a number of pre- and post-synaptic proteins, which are unaffected in the NeuroD2 null hippocampus. No comparisons reached statistical significance, n = 2 littermate pairs. Error bars represent standard error of the mean. Click here for file

Additional file 2 **NeuroD2 regulates the expression of SynCAM1 to -3 in the developing hippocampus**. **(A) **Immunoblots against SynCAM1, 2 and 3 from hippocampal lysates from WT and NeuroD2 null littermates at P14 and P21. Loading control is immunoblot against synaptophysin/p38. **(B) **Quantification of SynCAM1 expression. **(C) **Quantification of SynCAM2 expression. **(D) **Quantification of SynCAM3 expression. *P < 0.05, **P < 0.01, ***P < 0.001, *t*-test. Error bars represent standard error of the mean. N = 1 littermate pair at P14 and 2 littermate pairs at P21. Click here for file

Additional file 3 **Potential NeuroD2 binding sites upstream of PSD95 and SAP102**. Table listing sites within approximately 2 kb upstream of transcriptional start sites for PSD95 and SAP102 that conform to a consensus E-Box site to which NeuroD2 binds (CANNTG). Click here for file
