# Supplementary figures and images for: NeuroD2 regulates the development of hippocampal mossy fiber synapses
Source: Neural Dev. 2012 Feb 27;7:9. doi: 10.1186/1749-8104-7-9 (PMC3310804; doi:10.1186/1749-8104-7-9)

Additional File 1

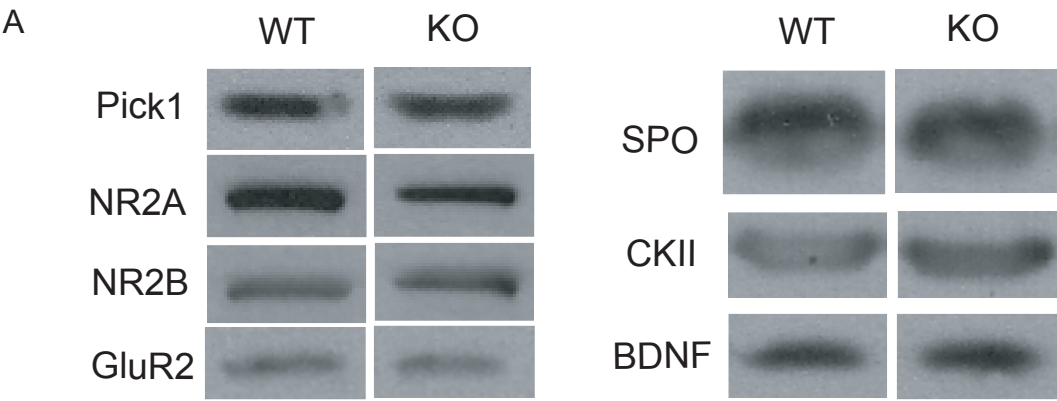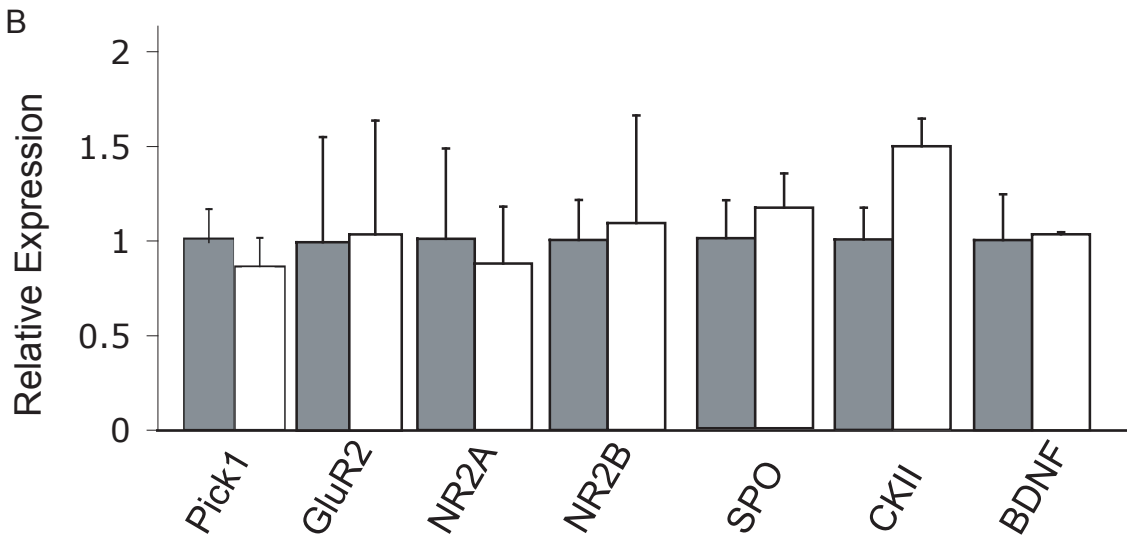

Supplement: Additional file 1 — Some synaptic proteins are unaffected in the NeuroD2 null hippocampus. (A) Representative immunoblots against hippocampal lysates from P21 WT and NeuroD2 null littermates. (B) Quantification of immunoblots against a number of pre- and post-synaptic proteins, which are unaffected in the NeuroD2 null hippocampus. No comparisons reached statistical significance, n = 2 littermate pairs. Error bars represent standard error of the mean. [file 1749-8104-7-9-S1.PDF]

# Additional File 2

A

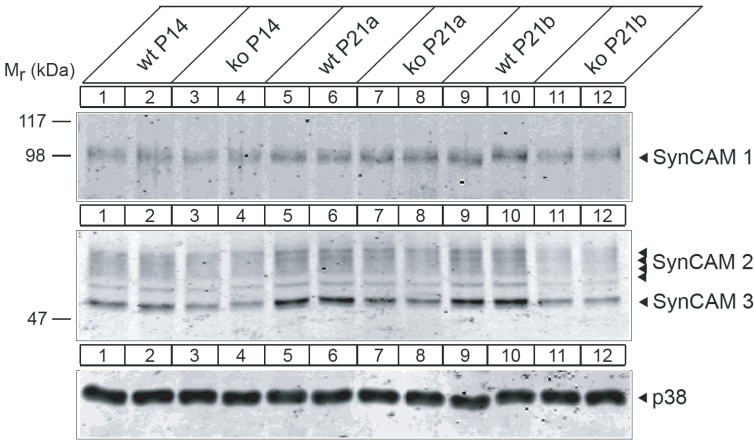

B

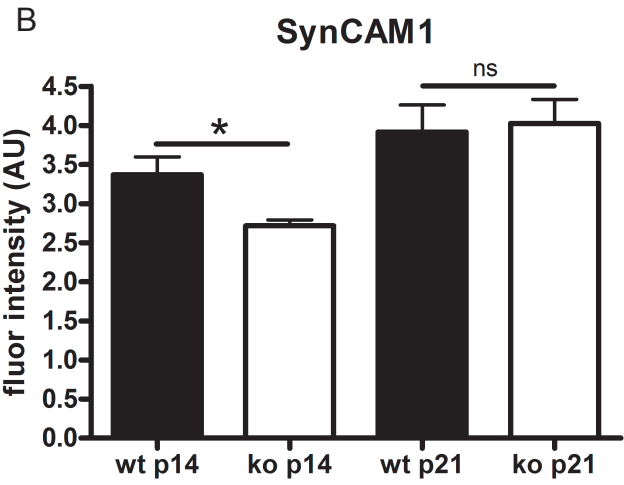

C

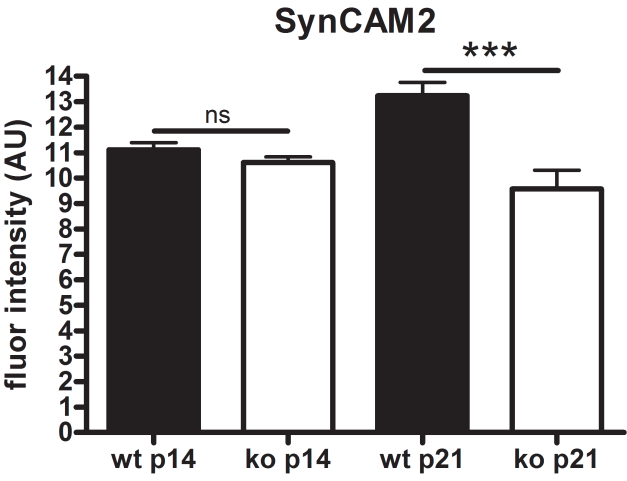

D

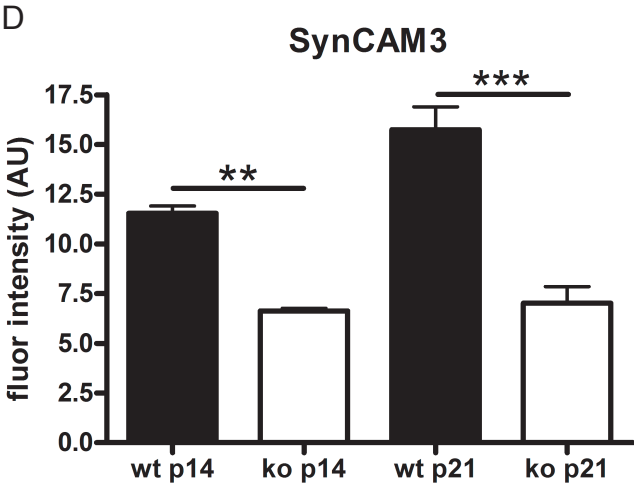

Supplement: Additional file 2 — NeuroD2 regulates the expression of SynCAM1 to -3 in the developing hippocampus. (A) Immunoblots against SynCAM1, 2 and 3 from hippocampal lysates from WT and NeuroD2 null littermates at P14 and P21. Loading control is immunoblot against synaptophysin/p38. (B) Quantification of SynCAM1 expression. (C) Quantification of SynCAM2 expression. (D) Quantification of SynCAM3 expression. *P < 0.05, **P < 0.01, ***P < 0.001, t-test. Error bars represent standard error of the mean. N = 1 littermate pair at P14 and 2 littermate pairs at P21. [file 1749-8104-7-9-S2.PDF]
