# Supplementary material for: NeuroD2 regulates the development of hippocampal mossy fiber synapses
Source: Neural Dev. 2012 Feb 27;7:9. doi: 10.1186/1749-8104-7-9 (PMC3310804; doi:10.1186/1749-8104-7-9)
Supplement: Additional file 3 — Potential NeuroD2 binding sites upstream of PSD95 and SAP102. Table listing sites within approximately 2 kb upstream of transcriptional start sites for PSD95 and SAP102 that conform to a consensus E-Box site to which NeuroD2 binds (CANNTG). [file 1749-8104-7-9-S3.PDF]

Additional File 3

PSD95		SAP102	
Sequence	Location	Sequence	Location
CAGCTG	-698 BP upstream	CAATTG	-374 BP upstream
CACTTG	-1510 BP upstream	CAGATG	-747 BP upstream
CAACTG	-1770 BP upstream	CACCTG	-1249 BP upstream
CATTTG	-1860 BP upstream	CAAATG	-1451 BP upstream
CAACTG	-2242 BP upstream	CATGTG	-1573 BP upstream
CACCTG	-2248 BP upstream	CATCTG	-1612 BP upstream
